# General anesthesia exposure in early life reduces the risk of allergic diseases: A nationwide population-based cohort study

**DOI:** 10.1097/MD.0000000000004269

**Published:** 2016-07-18

**Authors:** Ho-Chang Kuo, Ya-Ling Yang, Shu-Chen Ho, Mindy Ming-Huey Guo, Jyun-Hong Jiang, Ying-Hsien Huang

**Affiliations:** a Department of Pediatrics, Kaohsiung Chang Gung Memorial Hospital and Chang Gung University College of Medicine, Kaohsiung; b Kawasaki Disease Center, Kaohsiung Chang Gung Memorial Hospital, Taiwan University; c Department of Anesthesiology, Kaohsiung Chang Gung Memorial Hospital and Chang Gung University College of Medicine, Kaohsiung; d Department of Public Health, College of Health Sciences, Kaohsiung Medical University; e Department of Surgery, Kaohsiung Chang Gung Memorial Hospital and Chang Gung University College of Medicine, Kaohsiung, Taiwan.

**Keywords:** allergic disease, children, cohort study, general anesthesia

## Abstract

General anesthesia (GA) has been used for second line treatment strategy for status asthmaticus in pediatric patients. The association between GA in children and risk of followed-up allergic diseases is unclear. This study aims to assess the risk of allergic diseases after GA in children.

We did a nationwide retrospective cohort study by analyzing data from the National Health Insurance Research Database (NHIRD) in Taiwan. The subsequent risks for allergic diseases, including asthma (ICD-9: 493.X), allergic rhinitis (AR; ICD-9 CM code 477.X), and atopic dermatitis (AD; ICD-9-CM code 691.X), were compared between exposure to GA and none before 1 year of age throughout the follow-up period using the Cox proportional hazards model.

Insurance claims data for 32,742 children younger than 1 year old from all insured children in the NHIRD. Of those, 2358 subjects were exposed to GA; 414 and 1944 children exposed to mask and intubation ventilation, respectively, served as the study cohort, whereas the remaining 30,384 children made up the comparison cohort. Children in the GA group were at a lower risk of developing asthma, AR and AD, with adjusted hazard ratios of 0.67 (0.62–0.72, 95%CI), 0.72 (0.68–0.77, 95%CI), 0.60 (0.56–0.64, 95%CI), respectively.

Children who were exposed to GA in early life before 1 year of age had reduced risk of subsequently developing allergic diseases such as asthma, AD, and AR, when compared with general population.

## 1 Introduction

The increasing global incidences of allergic diseases, including asthma, allergic rhinitis (AR), and atopic dermatitis (AD), are important health problems for children.^[1,2]^ The growing prevalence of allergic disease has been triggered by a variety of factors, based on hygiene theory, such as the increased use of antibiotics, less contact with germs, decreased childhood infection rates, and improved environmental hygiene.^[3]^ An allergic disease rarely results in death and its related symptoms may affect the quality of life and cause economic burden.^[4]^ Therefore, understanding how allergic diseases develop and resolve through infancy and childhood is essential to clarify their pathophysiology.^[1]^

Most patients of asthma are effectively treated with standard therapy including β2-adrenergic agonists and corticosteroids.^[5]^ Nevertheless, volatile anesthetics have been used for refractory status asthmaticus in pediatric patients who do not respond to conventional therapy.^[6–8]^ The proposed mechanisms for volatile anesthetics include activation of β-adrenergic receptor, direct bronchial smooth muscle relaxation by inhibition of acetylcholine and histamine release as well as addition of sedation that reverse the underlying bronchoconstriction.^[7,9]^ Currently, the widespread and growing use of anesthesia in infants, defined as those less than 12 months of age, thus makes its safety an important health issue of interest to the public and government agencies. To the best of our knowledge, effect of general anesthesia (GA) exposure on the subsequent occurrence of asthma, AD, or AR among infants has yet to be determined. Thus, we conducted a population-based study related to GA exposure and subsequent risks of asthma, AR, and AD to establish the relationship between GA exposure in infants and the subsequent risk of developing an allergic disease.

## 2 Methods

Our study used data retrieved from the medical claims database of Taiwan's National Health Institute (NHI) program. The NHI program, which provides compulsory universal health insurance, was implemented in Taiwan on March 1, 1995 and has information about 99% of the 23.74 million residents living in Taiwan based on egalitarian ethical principles.^[10]^ Previous studies have described the details of the National Health Insurance Research Database (NHIRD),^[11,12]^ which contains medical information, including inpatient and outpatient care facilities, drug prescriptions, insurant sex, date of birth, date of visit or hospitalization, and diagnosis coded in the International Classification of Diseases, Ninth Revision, Clinical Modification (ICD-9-CM) format. Therefore, the information from the NHIRD database appears to be sufficiently complete, reliable, and accurate for use in epidemiological studies.^[10]^ In cooperation with the Bureau of NHI, the National Health Research Institute (NHRI) of Taiwan randomly sampled a representative database of 1,000,000 subjects from the entire NHI enrollees by means of a systematic sampling method for research purposes. There were no statistically significant differences in age, sex, and health care costs between the sample group and all enrollees, as reported by the NHRI.^[10,11]^

Children with a history of allergic disease, including asthma (ICD-9: 493.X), AR (ICD-9 CM code 477.X), and AD (ICD-9-CM code 691.X) before GA exposure and with incomplete data for age or sex at baseline were excluded from cohort study. We identified a cohort of 32,742 newborns in the period between January 1998 and December 2010, and those newborns were enrolled in this study. Information on GA exposure was extracted from the prescription database. The inhalation anesthetic agents are either sevoflurane or desflurance. Owing to more pleasant odor, sevoflurane is more readily accepted by patients, especially children.^[13]^

The subsequent risks for allergic diseases, including asthma, AR, and AD, were compared between exposure to GA and none before 1 year of age throughout the follow-up period using the Cox proportional hazards model. The observation period began on the index date and ended on the date of allergic disease diagnosis or on December 31, 2010. The length of follow-up was calculated for each patient diagnosed with one of the allergic diseases. The comorbidity of prematurity (ICD-9-CM 765) was collected for allergic diseases adjustment. The current study, using one of the aforementioned databases, was exempt from full review by Chang Gung Memorial Hospital's Institutional Review Board (IRB No.102-0364B) since the identification numbers of the patients in the database had been encrypted to protect their privacy.^[3]^

### 2.1 Statistical analysis

The person-years of follow-up for each case were calculated from the date of diagnosis of allergic disease to the date of death, or December 31, 2010. Incident rates were calculated by dividing the case number from allergic disease by the number of person-years of follow-up. Cox proportional hazard regression models adjusting for all potential confounders were used to estimate the relative magnitude of risk in relation to GA exposure. The participants were divided into GA exposure or none. We also divided GA exposure into mask anesthesia and intubation anesthesia. Hazard ratios (HRs) and their 95% confidence intervals (CIs) were calculated using patients with no exposure as reference. Analyses were performed using the SAS statistical package (version 9.3; SAS Institute Inc., Cary, NC). All statistical tests were 2-sided. A *P* value < 0.05 was considered statistically significant.

## 3 Results

The cohort contained 32,742 children younger than one year from all insured children in the NHIRD. Of those, 2358 subjects were exposed to GA; 414 and 1944 children exposed to mask and intubation ventilation, respectively, served as the study cohort, whereas the remaining 30,384 children made up the comparison cohort. As shown in Table 1, there were significant differences in the distribution of sex as well as prematurity, asthma, AR, and AD with or without GA exposure.

**Table 1:**
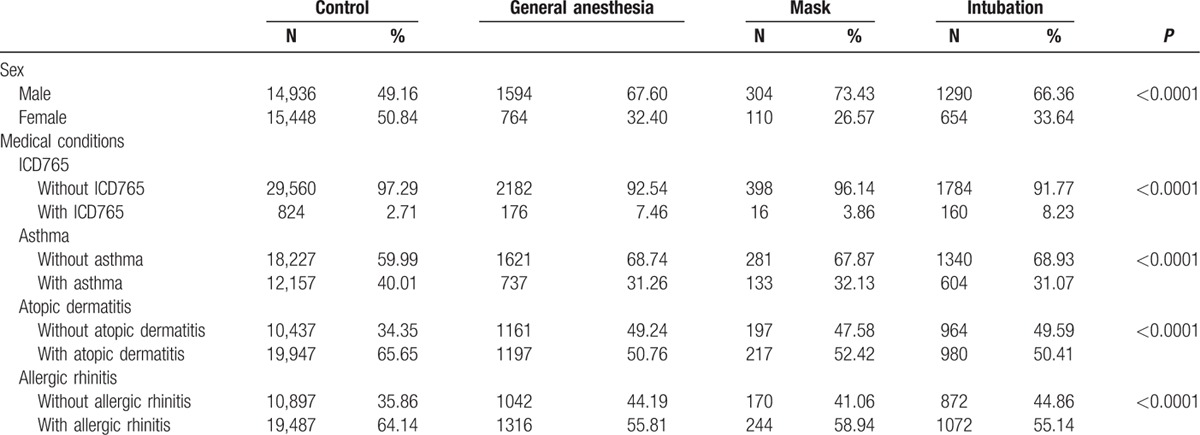
General characteristics of the study subjects.

### 3.1 Children with a history of GA exposure were at a lower subsequent risk of developing asthma

As shown in Table 2, asthma was found in 737 out of 2358 (31.2%) children with GA exposure and in 12,157 out of 30,384 (40.0%) children that had not suffered from this disease. Cox regression analysis showed that the HR for those children with GA exposure remained significant even after making adjustments for potential confounders including prematurity and sex (adjusted HR: 0.67; 0.62–0.72, 95% CI) throughout the 12-year follow-up period; that is, children with GA exposure were at a lower risk of subsequently developing asthma. Moreover, in GA exposure group, children who had mask and intubation ventilation showed no significant difference to each other and all showed a lower risk of subsequently developing asthma (adjusted HR: 0.68; 0.57–0.81, 95% CI and 0.67; 0.62–0.73, 95% CI, respectively).

**Table 2:**
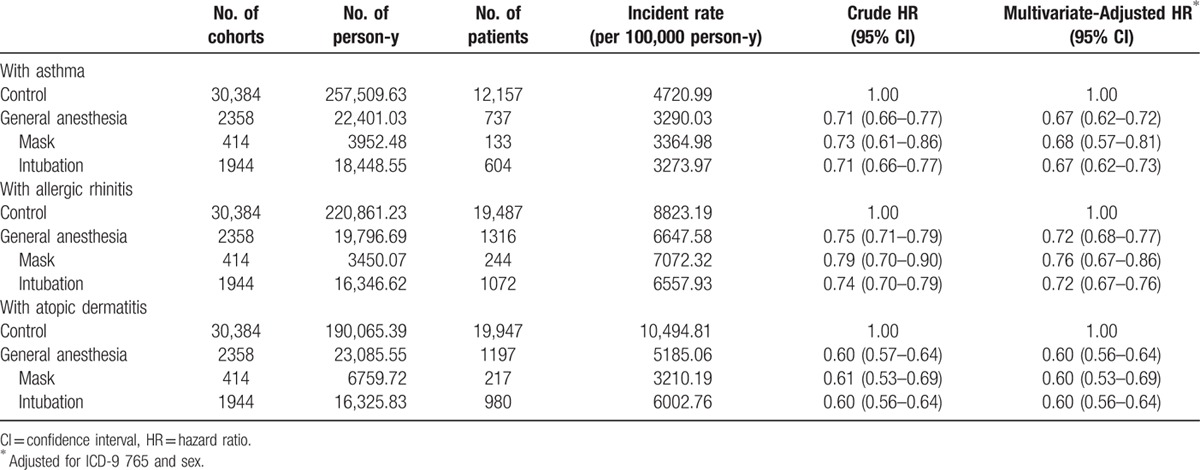
Risk of allergic diseases of children with a history of general anesthesia exposure.

### 3.2 Children with a history of GA exposure were at a lower subsequent risk of AR

As shown in Table 2, AR was found in 1316 out of 2358 (55.8%) children with GA exposure and in 19,487 out of 30,384 (64.1%) children that had not suffered from this disease. Cox regression analysis showed that the HR for those children with GA exposure remained significant even after making adjustments for potential confounders including prematurity and sex (adjusted HR: 0.72; 0.68–0.77, 95% CI) throughout the 12-year follow-up period; that is, children with GA exposure were at a lower risk of subsequently developing AR. Moreover, in GA exposure group, children who had mask and intubation ventilation showed no significant difference to each other and all showed a lower risk of subsequently developing AR (adjusted HR: 0.76; 0.67–0.86, 95% CI and 0.72; 0.67–0.76, 95% CI, respectively).

### 3.3 Children with a history of GA exposure were at a lower subsequent risk of AD

As shown in Table 2, AD was found in 1197 out of 2358 (50.7%) children with GA exposure and in 19,947 out of 30,384 (65.6%) children that had not suffered from this disease. Cox regression analysis showed that the HR for those children with GA exposure remained significant even after making adjustments for potential confounders including prematurity and sex (adjusted HR: 0.60; 0.56–0.64, 95% CI) throughout the 12-year follow-up period; that is, children with GA exposure were at a lower risk of subsequently developing AD. Moreover, in GA exposure group, children who had mask and intubation ventilation showed no significant difference to each other and all showed a lower risk of subsequently developing AD (adjusted HR: 0.60; 0.53–0.69, 95% CI and 0.60; 0.56–0.64, 95% CI, respectively).

## 4 Discussion

Children who were exposed to GA in early life of less than 1 year of age had decreased risk of developing allergic diseases including asthma, allergic rhinitis, and atopic dermatitis. From the literature review, this is the first study regarding GA and subsequent allergic diseases in childhood. Allergic diseases are mainly T-helper 2 (Th2) immune response dominant. Immune modulation in all allergic diseases has the common goal of decreasing the Th2 response, blocking critical Th2 cytokines, inhibiting Th2 cytokine synthesis, blocking critical Th2 effector molecules, inhibiting important cells in the Th2 response, and stimulating Th1 responses.^[14]^

One of the central theories regarding the development of allergy in children is called the “hygiene hypothesis”. This theory contends that during pregnancy Th2 immunity, which is mainly anti-inflammatory and prevents fetal rejection, is elevated, and in contrast Th1 immunity, which is related to inflammation and immune response to infections, is suppressed. Normally after birth, environmental factors, such as microbial exposure, promote Th1 whereas suppress Th2. However, modern environments lacking microbial or inflammatory exposure may cause an abnormal persistence of the Th2 immunity resulting in allergy.^[15]^ It is believed that GA may affect immune function either by exerting a direct effect on immune cells, or through the regulation of the pain and stress response caused by surgery.^[16]^ Although no previous reports have linked GA with the decrease of allergy later in life, it is possible that GA promotes inflammatory Th1 responses and decreases Th2 immunity which may be protective against the development of allergy. Supporting this hypothesis, prior studies have found that surgery-related postoperative release of pro-inflammatory cytokine IL-6 was increased in patients after spinal and GA.^[17]^ Increased levels of IL-2, which is produced by Th1 cells and required for Th1 differentiation, were found in patients anesthetized by GA.^[18]^ Interferon-gamma, another important Th1 cytokine, was found to be increased 24 hours after surgery in patients who were anesthetized by halothane and isoflurane.^[19]^

The inhalation anesthetic agents are either methyl-ethyl or isopropyl ether class.^[13]^ In the past decades, halothane has been gradually replaced by sevoflurane and desflurane because of their lower coefficients of blood solubility and decreased side effects.^[20]^ Moreover, due to pleasant odor that reduces scare in operation room^[13]^ and as numerous studies show that sevoflurane exerts a protective effect against bronchoconstriction,^[21,22]^ sevoflurane has several advantages in children. It is believed that GA may affect the regulatory balance of postoperative immune response.^[23]^ Sevoflurane could not only enhance the CD4+ lymphocytes in spleen in mice^[24]^ but also change peripheral blood leukocyte populations and antibody-producing capacity after either one or repeated exposures.^[25]^ Recently, it was also demonstrated that sevoflurane is involved in genetic methylation^[26]^ and histone acetylation^[27]^ in neonatal GA exposure in animals that may explain reducing allergic disease after GA exposure in younger stage of life.

Partially because of neonatal respiratory morbidity, preterm births are associated with an increased risk of asthma-like symptoms,^[28]^ and prematurity and low birth weight are significantly related to the decreased occurrence of AR in male conscripts.^[29]^ Furthermore, a low birth weight signifies a protective factor for the risk of AD.^[30]^ Ullemar et al^[31]^ reported that children born with a low gestational age or low birth weight have an increased risk of developing asthma. Moreover, the most common etiologies of received surgery are inguinal hernia,^[32]^ redundant prepuce, phimosis, tongue tie, and hydrocele that are male predominant and that is consistent with our results of GA group. As a result, prematurity or low birth weight may considerably influence the risk of subsequently developing allergic diseases; these 2 factors presented as ICD 765 and sex have been adjusted in this study. Additional studies are warranted to explore the mechanism about changes in the immune system caused by GA exposure and their long-term effects influencing the subsequent development of allergic diseases in humans.

## 5 Conclusions

This study is the first to investigate the allergic disease risk in children after having GA by using a population-based study, as well as the first to find that children who had early GA exposure before 1 year of age had reduced risk of subsequently developing allergic disease such as asthma, AD, and AR when compared with the general population.
